# Bilateral Lower Limb Deformity Correction in Osteogenesis Imperfecta Using a Non-elongating Rush Rod: A Pragmatic Approach

**DOI:** 10.7759/cureus.110147

**Published:** 2026-06-02

**Authors:** Thanikaivelan MK, Giriraj Harshavardhan, Mohammed Tavfiq, Sundar Suriyakumar, Selvamalaiyarasan S

**Affiliations:** 1 Department of Orthopaedics, Sri Ramachandra Institute of Higher Education and Research, Chennai, IND

**Keywords:** deformity correction, intramedullary fixation, long-bone bowing, lower limb deformity, non-telescopic rod, osteogenesis imperfecta, pediatric orthopedics, rush rod, skeletal dysplasia, sofield–millar osteotomy

## Abstract

Osteogenesis imperfecta is a genetic disorder caused by defects in type I collagen. Long-bone plastic bowing results from bone fragility, microfractures, deforming muscular forces, stress fractures, and malunion. The primary goal of treatment is deformity correction and stabilization to prevent recurrent fractures and progression. The management of long-bone deformities commonly involves corrective osteotomies with intramedullary stabilization. Although telescopic rods are widely preferred for their ability to accommodate skeletal growth, their availability and cost may limit their use in many centers. Therefore, we present a report of the management of severe bilateral femoral and tibial deformities in a child with osteogenesis imperfecta using non-telescopic Rush rods. A five-year-old boy presented with progressive deformities of the upper and lower limbs associated with an inability to walk independently following multiple fractures after trivial trauma. Clinical examination revealed blue sclerae, dentinogenesis imperfecta, short stature, thoracic kyphoscoliosis, and marked bowing deformities of the femur and tibia. Staged operative correction of bilateral lower limb deformities was performed. Multiple osteotomies were conducted at the apex of the deformities, followed by intramedullary stabilization of the femur and tibia using Rush rods. Postoperatively, hip spica immobilization was maintained for six weeks after each procedure, followed by gradual rehabilitation and radiographic monitoring. One instance of implant migration was noted during follow-up and addressed during the subsequent procedure. At one-year follow-up after the completion of staged correction, the patient was able to ambulate independently with maintained alignment, healed osteotomy sites, and satisfactory functional recovery. This case demonstrates that non-telescopic Rush rod fixation remains a useful option for deformity correction in osteogenesis imperfecta when telescopic systems are unavailable or technically unsuitable, and it highlights the surgical technique using Rush rod. Despite limitations related to skeletal growth and implant-related complications, satisfactory alignment and restoration of ambulation can be achieved with careful planning, meticulous surgical technique, and close follow-up.

## Introduction

Osteogenesis imperfecta is a rare genetic disorder and a form of skeletal dysplasia associated with collagen-related abnormalities, with an estimated prevalence of 1 in 15,000-20,000 individuals [1]. The disorder primarily affects type I collagen, the primary structural protein of the bone, skin, tendons, dentin, and sclera. Defects may be quantitative or qualitative, with qualitative abnormalities occurring more frequently. Clinically, children with osteogenesis imperfecta develop angular limb deformities secondary to fractures from minimal trauma, resulting from bone fragility, impaired remodeling, and progressive plastic deformation under physiological loading during growth [2]. These deformities significantly impair mobility and activities of daily living.

The most commonly used surgical technique is the Sofield-Millar osteotomy with fixation using non-elongating or elongating intramedullary devices [3,4]. Although elongating nails are considered superior because of their lower risk of refracture and deformity recurrence [5,6], their higher cost, limited availability, and technical demands may restrict their use in many centers. Consequently, non-elongating devices remain widely used in resource-limited settings [5,6]. Therefore, we present a case of bilateral lower limb deformity correction using a non-elongating Rush rod.

## Case presentation

A five-year-old boy presented with a 1.5-year history of difficulty in walking and deformities involving the upper and lower limbs. He had been apparently well until four years earlier, when he developed right leg pain following a trivial injury sustained while walking with support. He was diagnosed with a fracture and treated with native splinting for five cycles at 10-day intervals. One month later, he developed pain in both the upper limbs and the right thigh following a trivial fall and was treated conservatively with a plaster of Paris slab. Subsequently, he experienced multiple falls and continued conservative management. No history of abnormal bleeding, hearing loss, breathing difficulty, or chest pain was observed. The child was unable to stand or walk due to severe deformities and was carried around by his parents for activities of daily living.

**Figure 1:**
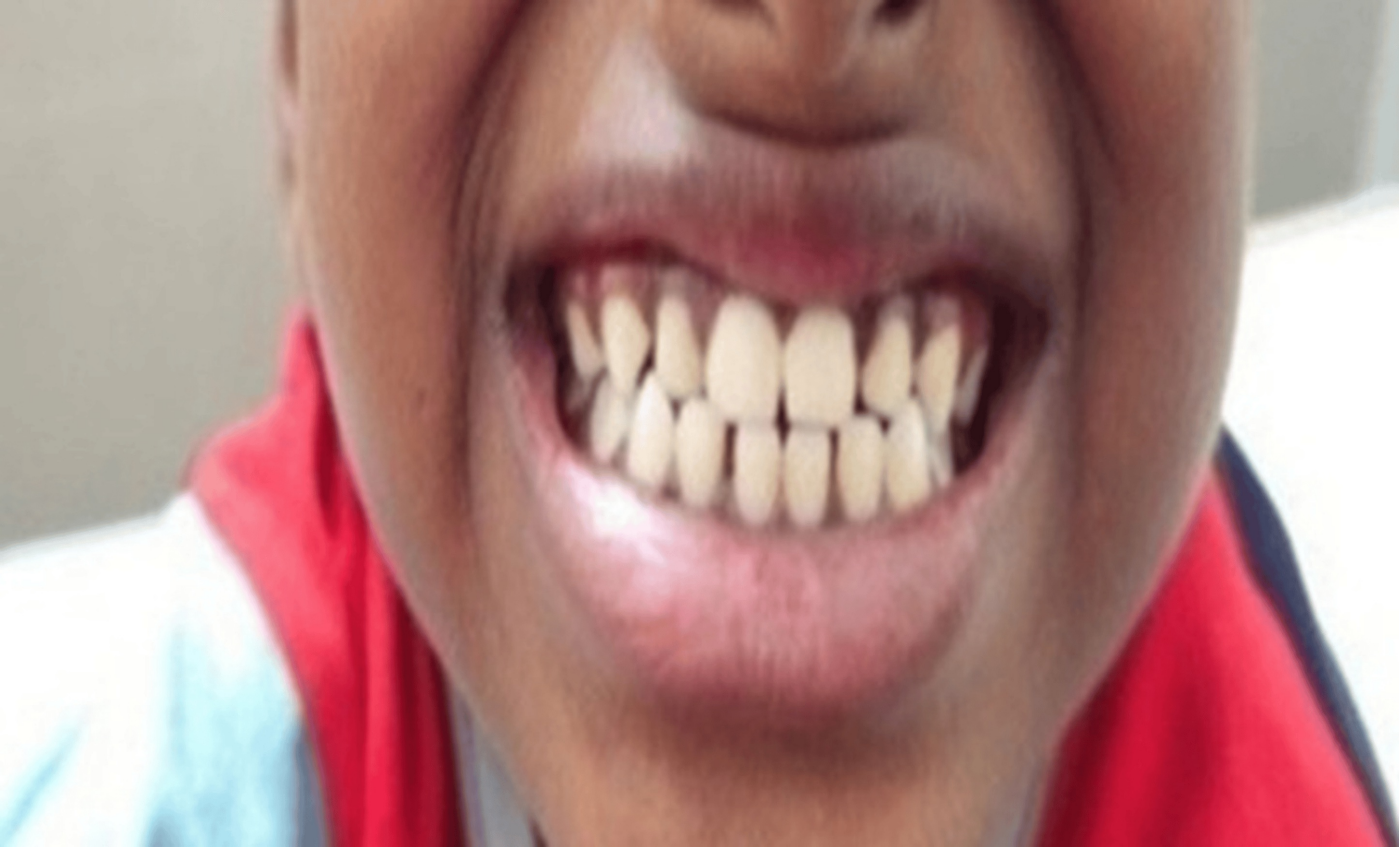
Clinical feature showing dentinogenesis imperfecta

**Figure 2:**
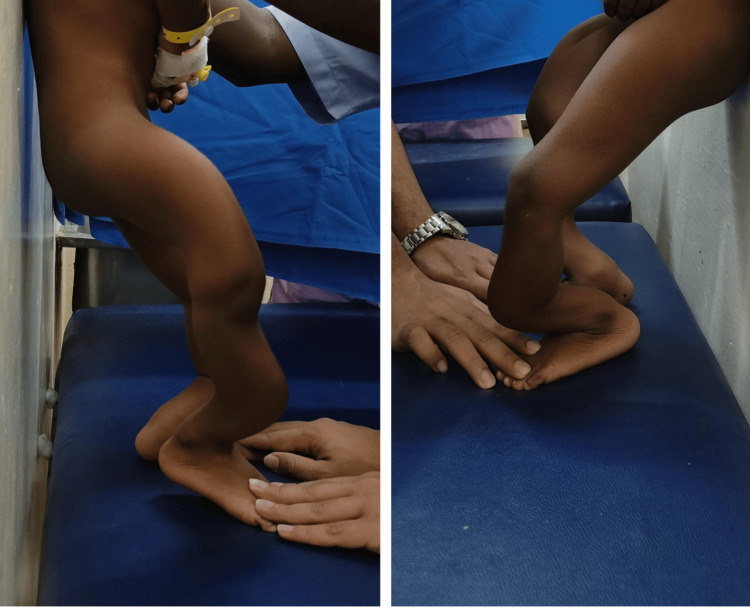
Lower limb deformity

**Figure 3:**
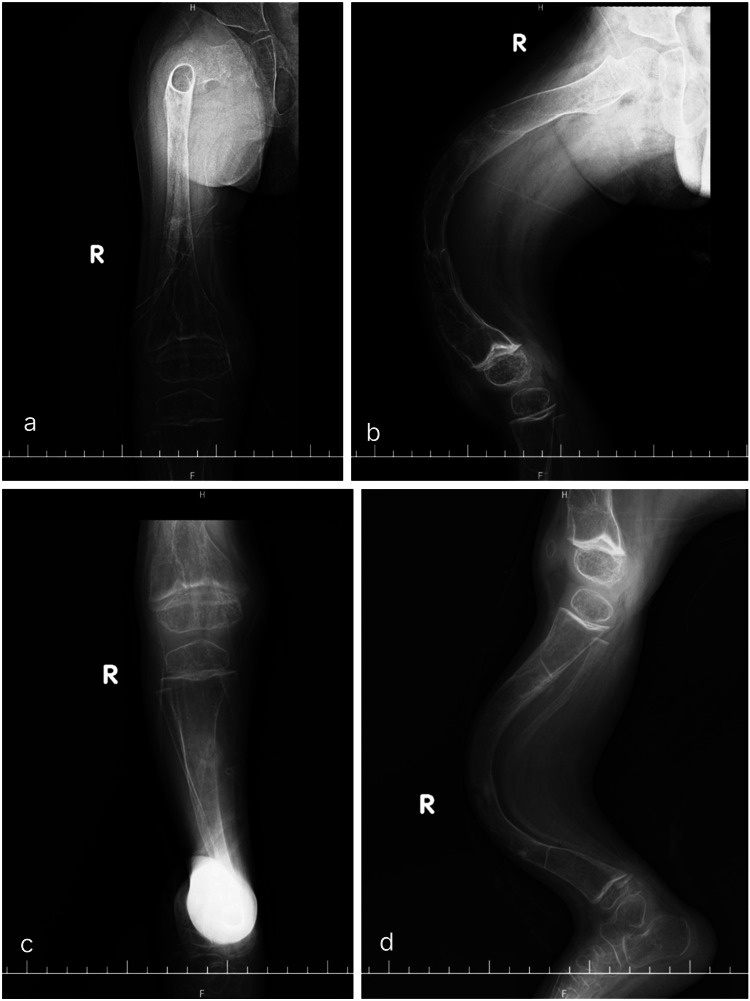
(a-d) Plain radiographs showing deformity of the right lower limb

**Figure 4:**
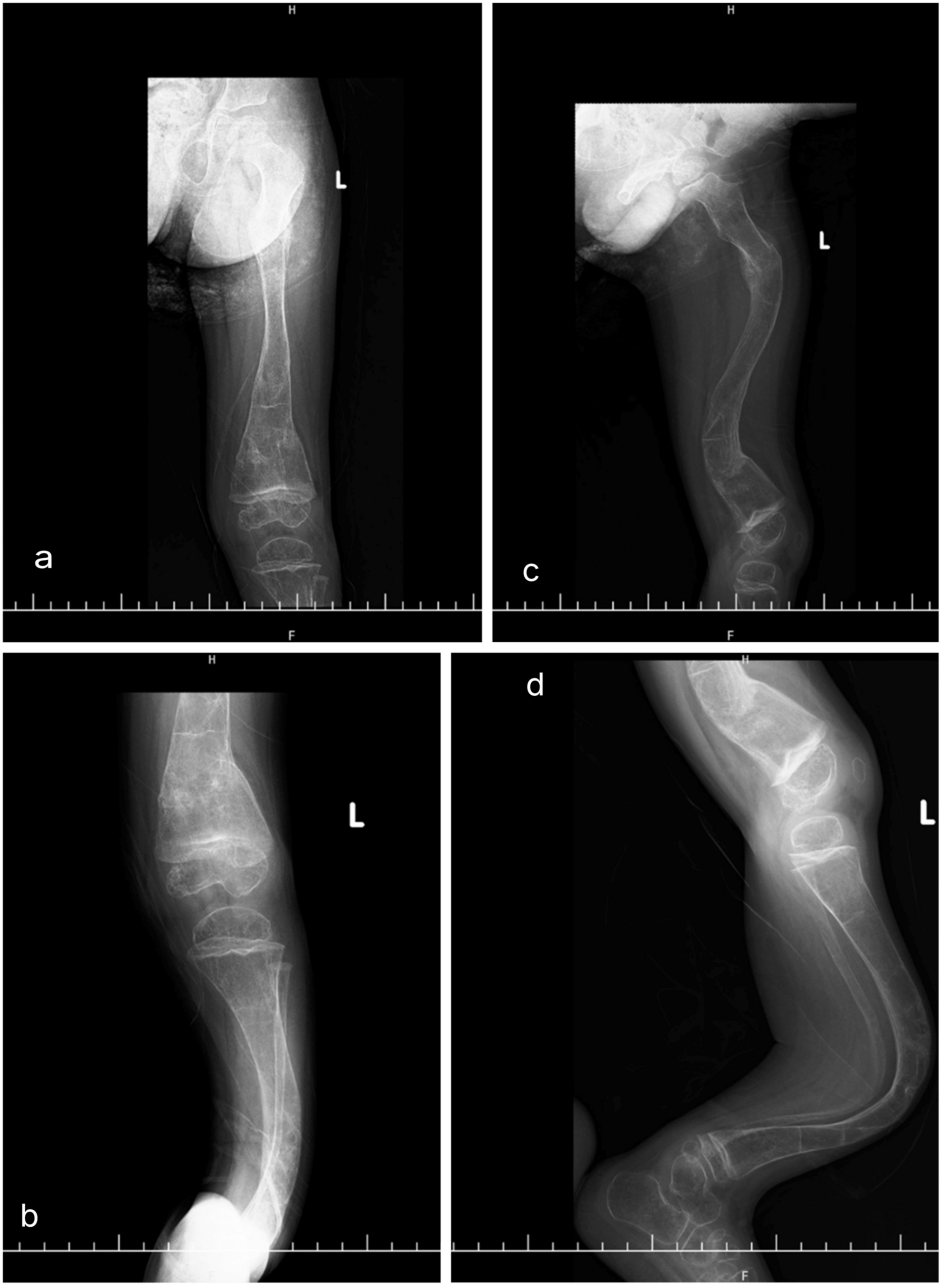
(a-d) Plain radiographs (anteroposterior and lateral views) showing deformity of the left lower limb

The face was triangular, and the fontanelle was closed. Dentition revealed dentinogenesis imperfecta (Figure 1). Short stature was noted. The spine showed thoracic kyphoscoliosis. The bilateral upper limb showed anterolateral bowing of the arm and forearm with visible muscle wasting. Palpable abnormal bony thickening was observed in the mid-arm and forearm. Bilateral lower limbs showed anterolateral bowing of the femur (Figures 2-4) and procurvatum of the middle third of the left tibia (Figure 4d), with a saber shin deformity. Similar bony thickening was also present at the mid-thigh and distal leg. No obvious limb length discrepancy was observed.

Metabolic bone evaluation demonstrated a serum calcium level of 10 mg/dL, phosphorus level of 5.7 mg/dL, alkaline phosphatase level of 316 U/L, and vitamin D level of 30.75 ng/mL. Clinical exome testing identified a homozygous WNT1 gene variant (c.1A>T; p.Met1?) associated with the osteogenesis imperfecta type XV phenotype, although the variant was classified as a variant of uncertain significance. The diagnosis was established based on the characteristic clinical features, radiographic findings, metabolic evaluation, and genetic testing results.

Pre-op planning and surgical technique

The child was initially planned for correction of bilateral femoral and tibial deformities to enable ambulation. A staged procedure for each limb was planned to reduce surgical morbidity. 

Right lower limb deformity correction was performed first. A tourniquet was not used, as combined correction of the tibial and femoral deformities was planned. The procedure was initiated with the tibia.

A medial parapatellar approach was initially used at the right knee to enable proximal Rush rod insertion. However, repeated attempts were unsuccessful. An anterior incision was then made over the tibial deformity site. Osteotomy was performed at the deformity site using a nibbler. A thin Rush rod was inserted retrograde from the osteotomy site through the medullary canal to identify the entry point. The Rush rod was found to track toward the lateral parapatellar region; therefore, a separate anterolateral incision was made to facilitate correct insertion of the Rush rod. Two osteotomies were performed in the bowed segment of the middle third of the tibia. The middle segment was also reamed using a Rush rod. A 3.5 x 170 mm Rush rod, shortened as required, was inserted proximally to stabilize the corrected tibia. Subsequently, femoral deformity correction was performed during the same procedure. An anterolateral approach was used to expose the femur at the site of maximal deformity. Two osteotomies were performed with a nibbler in the bowed segment of the middle third to achieve deformity correction. The entry point was identified by retrograde reaming of the proximal fragment after osteotomy using a thin Rush rod. An additional incision was made proximal to the greater trochanter to facilitate insertion of the Rush rod. The middle segment was also reamed. All reaming procedures were performed carefully using Rush rods, beginning with the smallest diameter. A 4.5 x 200 mm Rush rod was inserted proximally to stabilize the femur. 

**Figure 5:**
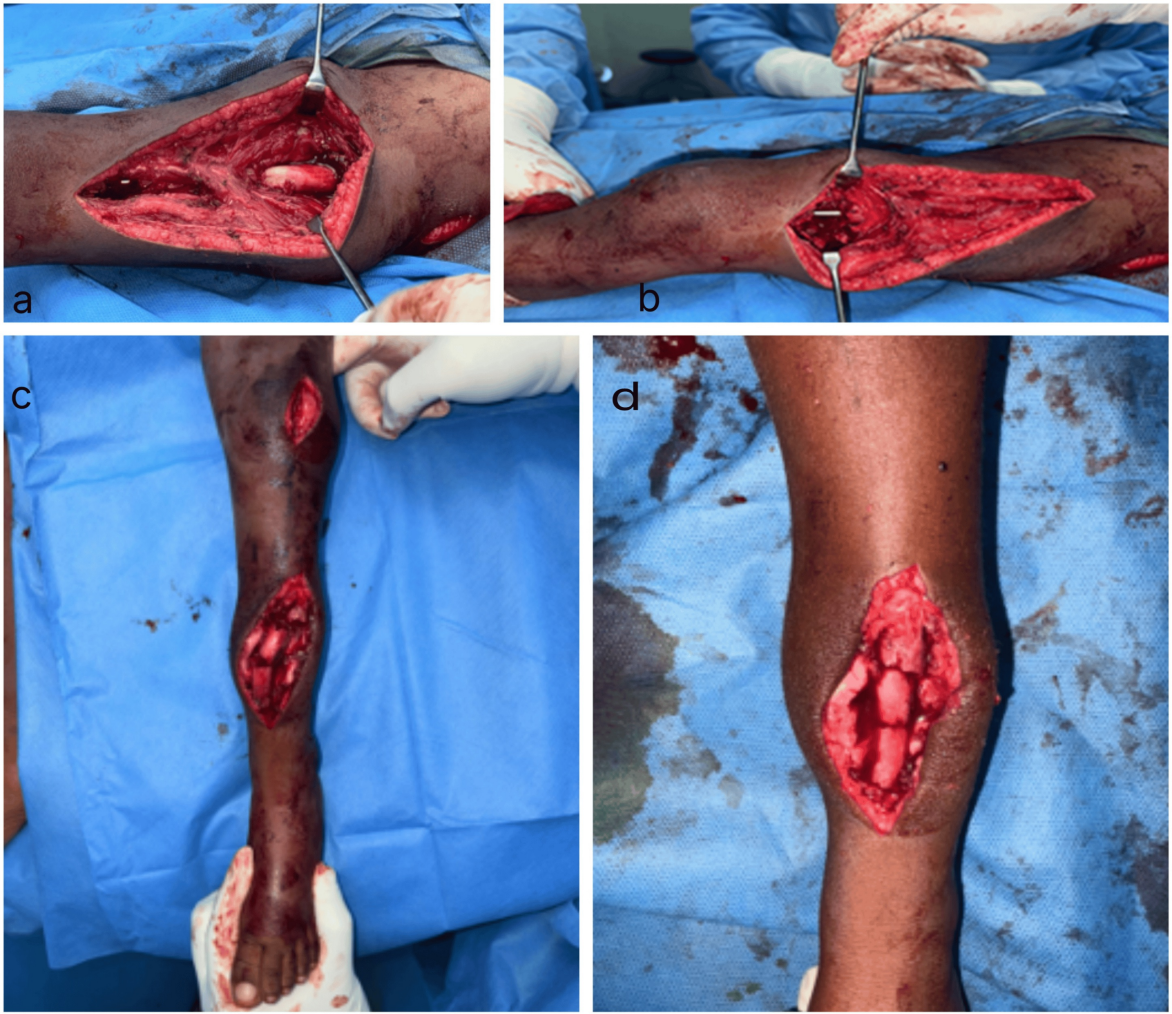
Intraoperative images following deformity correction of the (a, b) left femur and (c, d) left tibia, including the entry point

Left lower limb deformity correction was performed after an interval of eight months. A tourniquet was not used because correction of the tibia and femoral deformities was planned. The tibial procedure was performed first. An anterior incision was made over the site of the tibial deformity, and an osteotomy at the deformity site was performed using a nibbler. A thin Rush rod was passed retrograde from the osteotomy site through the canal to identify the entry point. The Rush rod was observed to exit in the medial parapatellar region; therefore, an anteromedial incision was performed over the knee to facilitate insertion of the Rush rod. Three osteotomies were performed in the bowed segment of the middle third of the tibia (Figures 5c, 5d). The middle segment was also reamed with the Rush rod. A 3.5 x 170 mm Rush rod, shortened as required, was inserted proximally to stabilize the corrected tibia. Subsequently, femoral deformity correction was performed during the same procedure. An anterolateral approach was used to expose the femur at the site of maximal deformity. Three osteotomies were performed with a nibbler in the bowed segment of the middle third to achieve deformity correction. The entry point was identified by retrograde reaming of the proximal fragment after osteotomy using a thin Rush rod. An additional incision was performed proximal to the greater trochanter to facilitate insertion of the Rush rod. The middle segment was also reamed. All reaming procedures were performed carefully using Rush rods, beginning with the smallest diameter. A 4.5 x 200 mm Rush rod was inserted proximally to stabilize the femur (Figures 5a, 5b). The Rush rod previously inserted into the right tibia eight months earlier had migrated proximally. The rod was advanced distally through the previously created anterolateral parapatellar entry incision. 

Following both procedures, a one-and-a-half hip spica was applied and maintained for six weeks. Serial radiographs were obtained to assess healing after removal of the hip spica at six weeks postoperatively.

Postoperative rehabilitation protocol

**Figure 6:**
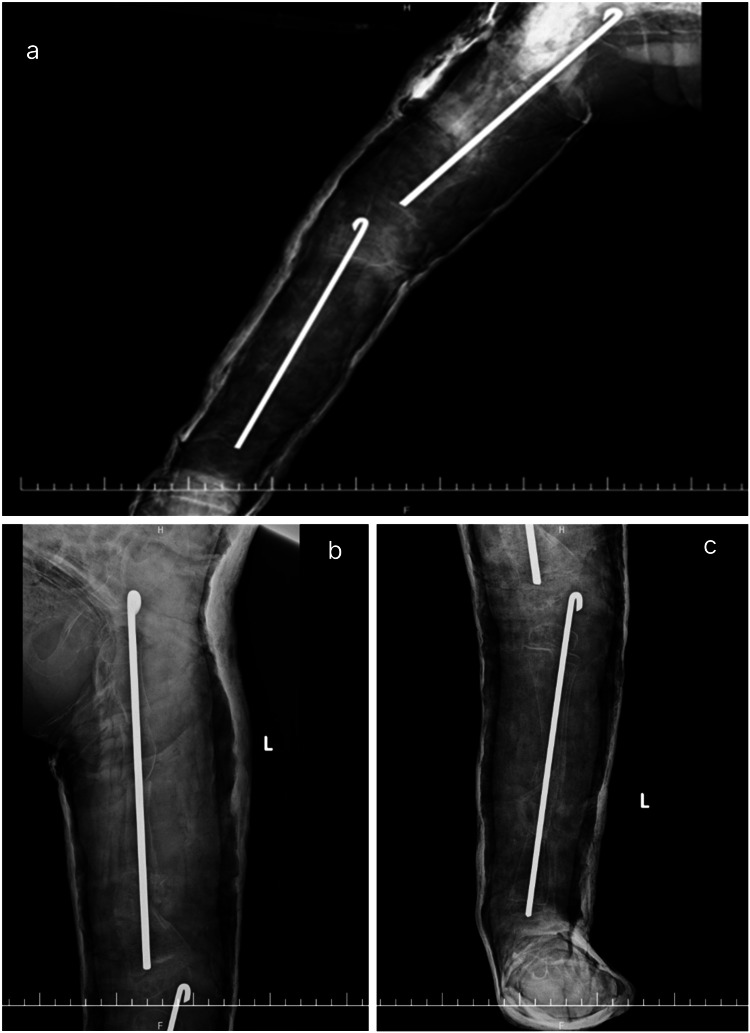
Postoperative radiograph after deformity correction of the right and left lower limbs

**Figure 7:**
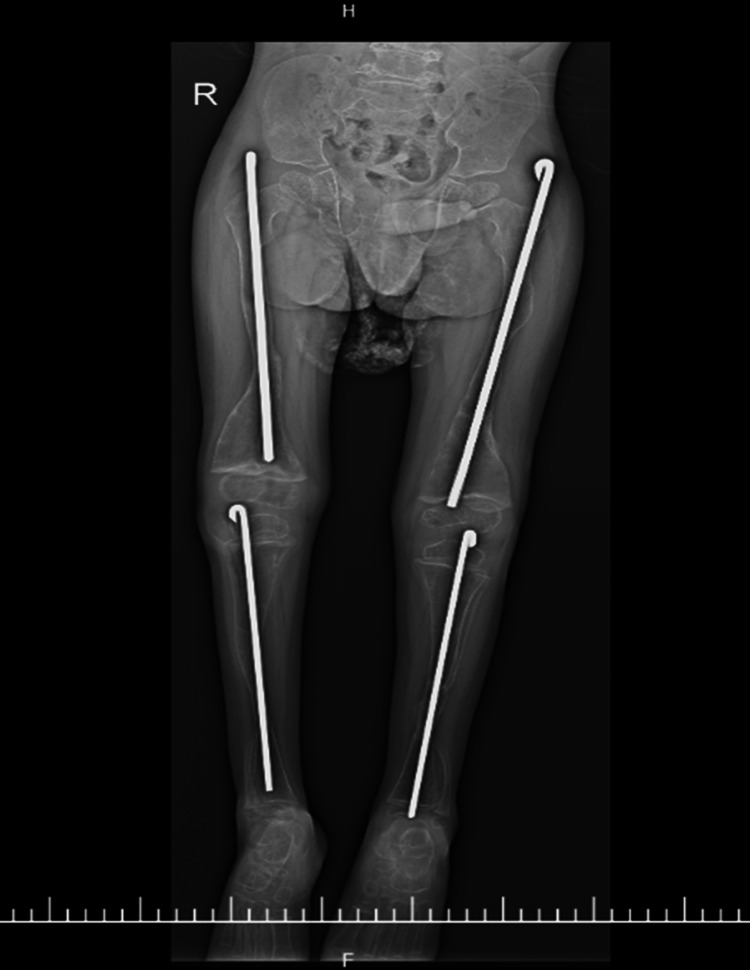
Radiograph at one-year postoperative follow-up

**Figure 8:**
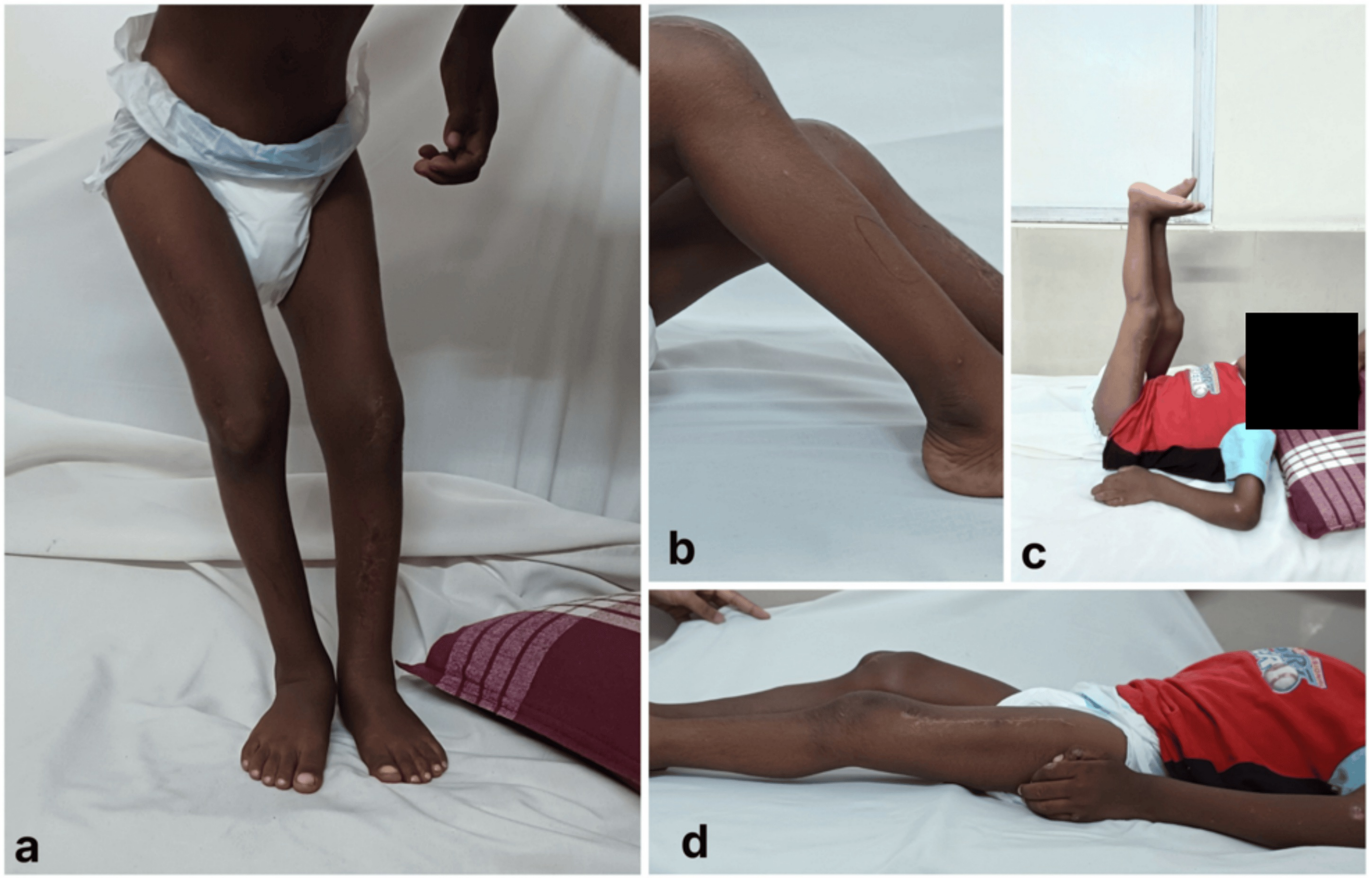
(a-d) Clinical photographs at one-year follow-up following deformity correction

The patient was maintained in a hip spica for six weeks (Figure 6). After spica removal, serial radiographs were obtained to assess healing. Gentle mobilization of the hip and knee joints was initiated. The child was initially mobilized with caregiver support, and weight-bearing was gradually progressed according to clinical and radiographic evidence of healing. No orthotic devices were used during the rehabilitation period. At one-year follow-up after the second procedure, the child was able to ambulate independently without significant difficulty. Radiographs obtained at one year (Figure 7) showed well-corrected deformities of the bilateral femora and tibiae, with intact nails, no evidence of migration, and healed osteotomies. The patient demonstrated satisfactory clinical alignment and independent ambulation at the final follow-up (Figure 8).

## Discussion

Osteogenesis imperfecta is a genetic disorder resulting from defects in type I collagen synthesis [1,7-9]. Plastic bowing of long bones occurs due to bone fragility, recurrent microfractures, deforming muscular forces, stress fractures, and malunion [10]. The primary goal of management is deformity correction and stabilization to prevent further fractures and progressive deformity [5]. Intramedullary fixation is preferred because plate fixation may create stress risers and increase the risk of subsequent fractures [4]. Deformity correction is commonly performed using the Sofield-Millar technique with elongating or non-elongating intramedullary devices. In this case, a Rush rod was used.

The Rush rod is a solid, straight intramedullary implant with a circular cross-section, beveled tip, and proximal hook. It functions as a condylocephalic fixation device based on the three-point fixation principle. Its elasticity permits controlled micromotion, thereby promoting callus formation and fracture healing. While elongating rods are currently preferred [7,11], the Rush rod remains advantageous owing to ease of insertion, cost-effectiveness, and availability. However, its inability to accommodate longitudinal bone growth remains a fundamental limitation. As the child grows, the nail no longer spans the entire length of the bone, leaving segments unprotected and predisposing to recurrent deformity and refracture. Another major drawback is the risk of implant-related complications. Implant migration is frequently reported due to poor epiphyseal purchase in osteoporotic bone and may lead to joint penetration or soft-tissue irritation [12]. In this study, proximal migration of the Rush rod in the right tibia was observed and later revised. In addition, loss of fixation, implant bending, and recurrence of deformity are commonly reported complications [5,12]. Other complications include growth-related deformity recurrence, fracture in unsplinted segments, and rod migration or extrusion. Therefore, regular follow-up is essential for early detection of these complications. Dual Rush rod fixation has been described as an alternative to elongating rods; however, it requires more experience and is technically difficult [6]. Because Rush rods do not accommodate skeletal growth, long-term follow-up is necessary to monitor for implant migration, recurrent deformity, refracture, and loss of fixation. Future procedures, including rod revision, replacement, or conversion to a telescopic system, may become necessary as skeletal growth progresses.

The fundamental advantage of telescopic nails lies in their ability to maintain stable fixation across the entire length of the bone throughout skeletal growth [6,13]. This reduces the incidence of stress risers and protects against recurrent deformity and fracture. However, telescopic systems are not without limitations. Their insertion is technically demanding and requires precise epiphyseal placement to ensure effective telescoping, and they are limited by minimum diameter requirements, making them unsuitable for very narrow medullary canals [11,14]. Since the telescopic rods are cannulated, they are prone to breakage as skeletal growth progresses [12]. Their higher cost and steep learning curve also limit widespread use. Other reported complications include nail migration, failure of the telescoping mechanism, refractures (rod and bone), joint penetration, and infection [6,13].

## Conclusions

Deformity correction using a Rush rod is an effective and practical option in selected young children with osteogenesis imperfecta, particularly when telescopic systems are unavailable, unaffordable, or technically unsuitable because of narrow intramedullary canals. With meticulous surgical technique, careful preoperative planning, staged deformity correction, and close postoperative follow-up, satisfactory alignment and functional outcomes are achieved. Although telescopic rods remain the preferred implants because they accommodate skeletal growth and reduce the risk of recurrent deformity, non-telescopic Rush rods continue to play an important role in resource-limited settings owing to their availability, lower cost, and technical simplicity. However, surgeons remain aware of complications such as implant migration, recurrence of deformity with growth, and the need for long-term follow-up and monitoring.

In the present case, staged bilateral femoral and tibial deformity correction with Rush rod stabilization resulted in restoration of the lower limb alignment, healing of osteotomy sites, and independent ambulation at one-year follow-up. This case demonstrates that satisfactory clinical and functional outcomes are achieved with non-elongating intramedullary fixation when performed with appropriate surgical planning and regular follow-up.
